# 38227 Specific and highly potent human monoclonal antibodies against SARS-CoV-2

**DOI:** 10.1017/cts.2021.459

**Published:** 2021-03-30

**Authors:** Mayara Garcia de Mattos Barbosa, Hui Liu, Daniel Huynh, Jeffrey L. Platt, Marilia Cascalho

**Affiliations:** 1 Department of Surgery, University of Michigan; 2 Departments of Surgery and Microbiology and Immunology, University of Michigan

## Abstract

ABSTRACT IMPACT: We devised a new method to produce highly potent SARS-CoV2-specific that can be used to treat severely ill patients with Covid-19. OBJECTIVES/GOALS: Neutralizing antibodies against SARS-CoV-2 are thought to offer the most immediate and effective treatment for those severely afflicted by Covid-19. We devised an approach for rapid and efficient generation of human monoclonal antibodies with neutralizing activity against SARS-CoV-2. METHODS/STUDY POPULATION: SARS-CoV-2 S1 spike protein-specific memory B cells were isolated from 12 subjects recovering from infection with that virus. Paired end single index sequencing was performed using up to 10,000 antigen-specific B cells per subject. Antigen-specific B cell clones were identified by unique diversity and joining gene V(D)J rearrangements and the CDR3 regions. VH and VL regions were cloned and the products expressed in 293T/17 cells to generate spike-specific human monoclonal antibodies. RESULTS/ANTICIPATED RESULTS: Forty-three human monoclonal antibodies were produced. Every monoclonal antibody so generated neutralized viruses pseudotyped with Spike protein of the Wuhan-1 strain. Eighteen monoclonal antibodies neutralized pseudotyped viruses with half-maximal inhibitory concentration (IC50s) between 1 pg/mL and 1 ng/mL (6.7 x 10E-15 M to 6.7 x 10E-12 M), exceeding by 10-100-fold the potency of previously reported anti-SARS-CoV-2-neutralizing monoclonal antibodies. Eight monoclonal antibodies neutralized viruses pseudotyped with mutant spike proteins previously identified in clinical isolates, including receptor binding domain mutants and the C-terminal D614G mutant with IC50<6.7 x10E-12M. DISCUSSION/SIGNIFICANCE OF FINDINGS: We show that SARS-CoV-2 evokes high affinity B cell responses. Some B cells produce antibodies that are broadly neutralizing; others produce strain-specific antibodies. However, antigenic variants that would potentially escape control by immunity or vaccination were nonetheless identified.

